# Production of lipopeptide biosurfactants by *Bacillus atrophaeus* 5-2a and their potential use in microbial enhanced oil recovery

**DOI:** 10.1186/s12934-016-0574-8

**Published:** 2016-10-03

**Authors:** Junhui Zhang, Quanhong Xue, Hui Gao, Hangxian Lai, Ping Wang

**Affiliations:** 1 College of Natural Resources and Environment, Northwest A & F University, 3 Taicheng Road, 712100 Yangling, China; 2 College of Earth Sciences and Resources, Chang’an University, 710055 Xi’an, China

**Keywords:** Microbial enhanced oil recovery, Biosurfactant, *Bacillus atrophaeus*, Surface tension, Crude oil removal

## Abstract

**Background:**

Lipopeptides are known as promising microbial surfactants and have been successfully used in enhancing oil recovery in extreme environmental conditions. A biosurfactant-producing strain, *Bacillus atrophaeus* 5-2a, was recently isolated from an oil-contaminated soil in the Ansai oilfield, Northwest China. In this study, we evaluated the crude oil removal efficiency of lipopeptide biosurfactants produced by *B. atrophaeus* 5-2a and their feasibility for use in microbial enhanced oil recovery.

**Results:**

The production of biosurfactants by *B. atrophaeus* 5-2a was tested in culture media containing eight carbon sources and nitrogen sources. The production of a crude biosurfactant was 0.77 g L^−1^ and its surface tension was 26.52 ± 0.057 mN m^−1^ in a basal medium containing brown sugar (carbon source) and urea (nitrogen source). The biosurfactants produced by the strain 5-2a demonstrated excellent oil spreading activity and created a stable emulsion with paraffin oil. The stability of the biosurfactants was assessed under a wide range of environmental conditions, including temperature (up to 120 °C), pH (2–13), and salinity (0–50 %, w/v). The biosurfactants were found to retain surface-active properties under the extreme conditions. Additionally, the biosurfactants were successful in a test to simulate microbial enhanced oil recovery, removing 90.0 and 93.9 % of crude oil adsorbed on sand and filter paper, respectively. Fourier transform infrared spectroscopy showed that the biosurfactants were a mixture of lipopeptides, which are powerful biosurfactants commonly produced by *Bacillus* species.

**Conclusions:**

The study highlights the usefulness of optimization of carbon and nitrogen sources and their effects on the biosurfactants production and further emphasizes on the potential of lipopeptide biosurfactants produced by *B. atrophaeus* 5-2a for crude oil removal. The favorable properties of the lipopeptide biosurfactants make them good candidates for application in the bioremediation of oil-contaminated sites and microbial enhanced oil recovery process.

## Background

Biosurfactants are a heterogeneous group of surface-active molecules produced by microorganisms, such as bacteria, fungi, and yeasts [1]. The molecular structures of biosurfactants include a hydrophilic moiety, comprising an amino acid or peptide, anions or cations, mono-, di-, or polysaccharides; and a hydrophobic moiety of unsaturated, saturated, or hydrocarbon fatty acids [2]. Therefore, biosurfactants reduce surface tension and interfacial tension in both aqueous solutions and hydrocarbon mixtures and form micelles and microemulsions between the two phases [2, 3]. Such surface properties make biosurfactants good candidates for enhancing oil recovery [4, 5]. Bailey et al. [6] reported that a biosurfactant flooding process, using a low concentration (35–41 ppm) of biosurfactants produced by *Bacillus mojavensis* strain JF-2, resulted in high oil recovery, of up to 35–45 %. In recent years, an increase in concern about environmental protection has caused the development of cost-effective bioprocesses for biosurfactants production [7]. The use of biosurfactants that have a comparable enhanced oil recovery performance is preferable [4].

Based on the types of biosurfactant-producing microbial species and the nature of their chemical structures, biosurfactants can be roughly divided into four groups: lipopeptides and lipoproteins, glycolipids, phospholipids, and polymeric surfactants [8]. Among these four groups, the best-known compounds are lipopeptides, produced by *Bacillus* species, and glycolipids, produced by *Pseudomonas* species [9]. In general, mixtures of cyclic lipopeptides are built from variants of heptapeptides and hydroxy fatty acid chains [8], while glycolipids are mixtures of rhamnolipid homologs, composed of one or two rhamnose molecules linked to one or two hydroxy fatty acid chains [10]. The two types of biosurfactants improve oil recovery by reducing the interfacial tension and altering the wettability of reservoir rock [11]. Glycolipids have been extensively studied in microbial enhanced oil recovery (MEOR) experiments and lipopeptides, such as surfactin and iturins, have also been found effective in similar studies [12]. Surfactin is known as a powerful microbial surfactant with high surface activities and has been successfully used in enhancing oil recovery [12–14].

Biosurfactants MEOR represents a promising method to recover a substantial proportion of the residual oil from marginal oil fields [15, 16]. Biosurfactants can be implemented in two ways: they can be produced either ex situ to be injected into the reservoir or in situ by indigenous or injected microorganisms [15]. The first approach involves the production of biosurfactants above ground by fermentation and therefore requires expensive equipment, including bioreactor and purification systems [16]. The second method is more favorable from an economic point of view, but the indigenous microorganisms need to be identified and their capacity to grow and produce sufficient amounts of biosurfactants in oil reservoirs assessed. Unfortunately, this process cannot be completely manipulated and this places limitations on the reservoirs where microorganisms can be used for in situ treatment [17].

There have been several successful studies into the application of biosurfactants during in situ or ex situ field tests [12]; Recently, a field study demonstrated that approximately nine times the minimum concentration of biosurfactants required to mobilize oil was produced in situ by a consortium of *Bacillus* strains, resulting in the recovery of substantial amount of oil entrapped in the limestone reservoir of the Bebee field, Pontotoc City, Oklahoma, USA [18]. Additionally, a study tested the interaction of biosurfactant produced by *B. subtilis* W19 with porous media in coreflooding experiments as a tertiary-recovery stage. *B. subtilis* W19 showed high potential of oil extraction during ex situ MEOR applications in which a total of 23 % of residual oil was extracted produced after biosurfactant and concentrated-biosurfactant injection [19]. The main drawbacks of lipopeptide biosurfactants for MEOR are low yields and high production costs [20].

The aims of this work were to: (1) improve lipopeptide biosurfactant production yields, through selection of an appropriate bacteria strain and optimization of the carbon and nitrogen sources in the culture media; (2) characterize the biosurfactants produced by the bacteria selected; (3) assess the surface activities and potential of the biosurfactants produced; and (4) determine the feasibility for their use in MEOR.

## Results and discussion

### Effect of carbon source on biosurfactant production

*Bacillus atrophaeus* 5-2a was able to grow and produce biosurfactants utilizing all of the carbon sources tested, except paraffin (Table 1). When liquid paraffin was the sole carbon source, there was some growth, but it was lower than that observed with the water-soluble carbon sources (Table 1). Several studies have shown, with different *Bacillus* strains, that if hydrocarbons (including *n*-hexadecane and paraffin) are the only carbon source, bacterial growth and biosurfactant production is either completely inhibited [21, 22], or severely limited [16].

**Table 1:** Dry cell weight (g L^−1^), crude biosurfactant yield (g L^−1^), oil spreading (cm), emulsification index (%), and surface tension (mN m^−1^) obtained for *Bacillus atrophaeus* 5-2a grown in mineral salt solution with different carbon sources at 30 °C for 5 days

Carbon source	Dry cell weight (g L^−1^)	Crude biosurfactant yield (g L^−1^)	Oil spreading (cm)	Emulsification index (%)	Surface tension (mN m^−1^)
Brown sugar	0.56 ± 0.0071c	0.95 ± 0.071b	18.4 ± 0.10b	61.81 ± 0.98a	26.12 ± 0.085c
Sucrose	0.37 ± 0.028e	0.74 ± 0.085c	18.1 ± 0.16c	56.76 ± 0.25c	26.32 ± 0.035b
Glucose	0.33 ± 0.021e	0.53 ± 0.071d	17.2 ± 0.12e	58.34 ± 0.33b	26.38 ± 0.035b
Maltose	0.86 ± 0.035a	0.82 ± 0.085bc	18.2 ± 0.10bc	54.80 ± 0.18d	26.11 ± 0.028c
Starch	0.51 ± 0.014cd	0.71 ± 0.071c	17.7 ± 0.12d	56.85 ± 0.13c	26.39 ± 0.099b
Mannitol	0.48 ± 0.0071d	1.11 ± 0.042a	19.6 ± 0.071a	54.11 ± 0.085d	25.82 ± 0.028d
Glycerol	0.80 ± 0.014b	0.72 ± 0.028c	17.8 ± 0.12d	57.43 ± 0.14bc	26.32 ± 0.057b
Paraffin	0.14 ± 0.028f	0.06 ± 0.028e	8.2 ± 0.16f	0.00 ± 0.00e	40.49 ± 0.057a

Values are presented as the mean ± standard deviation (*n* = 3). Different superscript letters within a column indicate significant differences (*P* < 0.05) by Duncan’s multiple range test

The highest dry cell weights (0.86 and 0.80 g L^−1^, respectively) were obtained using maltose and glycerol as the carbon source. The lowest surface tension (ST) of the culture supernatant (25.82 mN m^−1^) was obtained when mannitol was the sole carbon source. However, the other carbohydrate sources tested also decreased ST in the range of 26.11–26.39 mN m^−1^, except paraffin. Glucose, molasses, and palm oil have been found to be the best carbon sources for the growth of *Bacillus* isolates [9, 14]. Additionally, *Bacillus* strains were reported to grow utilizing glycerol and sucrose as the sole carbon sources and the STs of the culture broths were 27.1 and 27.9 mN m^−1^, respectively [16, 23].

The highest emulsifying activity of the culture was obtained using brown sugar as the carbon source (61.81 %), followed by glucose (58.34 %), glycerol (57.43 %), starch (56.85 %), sucrose (56.76 %), maltose (54.80 %) and mannitol (54.11 %). Raw glycerol from the biodiesel industry has previously been identified as a potential low-cost carbon source for biosurfactant production, with an emulsification efficiency of 67.6 % against crude oil [24]. Furthermore, Al-Wahaibi et al. [14] found that the biosurfactants produced by *Bacillus subtilis* B30 had a high emulsifying activity against various hydrocarbons when glucose and molasses were used as the carbon sources.

The amount of biosurfactants produced varied from 0.53 to 1.11 g L^−1^ and the diameter of oil spreading ranged from 17.2 to 19.6 cm, depending on the carbon source used (Table 1). The highest crude biosurfactant yield and diameter of oil spreading were obtained when mannitol was used as the carbon source. In the second place, the crude biosurfactant yield and diameter of oil spreading reached 0.95 g L^−1^ and 18.4 cm, respectively, with brown sugar as the carbon source. These results are in agreement with the ST results obtained for *B. atrophaeus* 5-2a, but in contrast with the highest emulsifying activity (achieved with brown sugar). This indicates that various types of biosurfactants with different properties were synthesized by this strain, depending on the carbon source used.

### Effect of nitrogen source on biosurfactant production

*Bacillus atrophaeus* 5-2a was able to utilize all of the nitrogen sources tested (Table 2); growth was accompanied with biosurfactant production. The highest dry cell weight (0.78 g L^−1^) was obtained using urea as the nitrogen source in the culture. For biosurfactant production, the nitrogen source can be inorganic (e.g., NaNO_3_, NH_4_Cl, (NH_4_)_2_SO_4_, NH_4_NO_3_ or urea) or organic (e.g., beef extract, tryptone, or yeast extract). In previous studies, some *B. subtilis* strains could not use (NH_4_)_2_SO_4_ or KNO_3_ for microbial growth or biosurfactant production; however, they could use NaNO_3_, NH_4_NO_3_ or KNO_3_ [21, 25]. In this study, the fact that *B. atrophaeus* 5-2a could grow and produce biosurfactants using all of the nitrogen sources tested indicates that it is more competitive than previous *Bacillus* strains tested for industrial applications.

**Table 2:** Dry cell weight (g L^−1^), crude biosurfactant yield (g L^−1^), oil spreading (cm), emulsification index (%), and surface tension (mNm^−1^) obtained for *Bacillus atrophaeus* 5-2a grown in mineral salt solution with different nitrogen sources at 30 °C for 5 days

Nitrogen source	Dry cell weight (g L^−1^)	Crude biosurfactant yield (g L^−1^)	Oil spreading (cm)	Emulsification index (%)	Surface tension (mN m^−1^)
Beef extract	0.64 ± 0.021e	0.47 ± 0.014c	16.5 ± 0.10f	59.50 ± 0.34c	27.64 ± 0.028b
Peptone	0.87 ± 0.057d	0.66 ± 0.028ab	18.8 ± 0.10b	59.47 ± 0.36c	26.65 ± 0.057d
Corn steep liquor	0.63 ± 0.028e	0.42 ± 0.085c	14.2 ± 0.16 g	10.41 ± 0.57d	29.51 ± 0.035a
Urea	0.99 ± 0.028c	0.78 ± 0.028a	19.2 ± 0.10a	60.54 ± 0.38ab	26.43 ± 0.021e
NH_4_Cl	1.41 ± 0.014a	0.55 ± 0.071bc	16.9 ± 0.10e	59.34 ± 0.18c	27.64 ± 0.014b
(NH_4_)_2_SO_4_	1.22 ± 0.014b	0.66 ± 0.085ab	17.2 ± 0.12d	61.16 ± 0.25a	27.42 ± 0.092c
NaNO_3_	0.85 ± 0.0071d	0.73 ± 0.042a	17.6 ± 0.12c	61.23 ± 0.59a	27.38 ± 0.099c
KNO_3_	0.47 ± 0.014f	0.53 ± 0.099bc	16.7 ± 0.16e	60.14 ± 0.19bc	27.60 ± 0.057b

Values are presented as the mean ± standard deviation (*n* = 3). Different superscript letters within a column indicate significant differences (*P* < 0.05) by Duncan’s multiple range test

The lowest ST, which corresponded to the highest crude biosurfactant yield and the biggest the diameter of oil spreading, was obtained when urea was used as the sole nitrogen source (26.43 mN m^−1^). The other nitrogen sources tested also offered good results in terms of ST (26.65–29.51 mN m^−1^), crude biosurfactant yield (0.42–0.73 g L^−1^) and diameter of oil spreading (14.2–19.2 cm) for the culture supernatant. These results agree with Makkar and Cameotra [25] who reported that the maximum amount of biosurfactant, and ST values between 29 and 29.5 mN m^−1^, were produced by a thermophilic *B. subtilis* when urea or nitrate ions were supplied as the nitrogen sources.

The highest emulsifying activity was observed when (NH_4_)_2_SO_4_ and NaNO_3_ were used (61.16 and 61.23 %, respectively), followed by KNO_3_ (61.14 %), urea (60.54 %), beef extract (59.50 %), peptone (59.47 %) and NH_4_Cl (59.34 %). This is in agreement with the results reported by Dastgheib et al. [22], in which sodium nitrate was the best substrate for emulsifier production, followed by urea, yeast extract and peptone.

Among all of the carbon and nitrogen sources tested, brown sugar and urea were found to be the most suitable carbon and nitrogen sources regarding the amounts of crude biosurfactant, diameter of oil spreading, emulsifying activity and ST. They are also inexpensive and easily available, making their potential application in MEOR economically feasible. Therefore, brown sugar and urea were selected as the carbon and nitrogen sources for the remaining experiments.

### Comparison of the optimal media for biosurfactant production

The potential use of *Bacillus* strains for biosurfactant production has been widely described in the literature [14, 16, 20]. To the authors’ knowledge, however, no studies have examined the production of biosurfactants by *B. atrophaeus*. In the study, *B. atrophaeus* 5-2a demonstrated a higher ability to produce biosurfactants in the BB medium than the BU medium. Its production of biosurfactants in the BU medium was assessed to ascertain its potential to ferment cheaper raw materials (i.e., urea and brown sugar).

#### Biosurfactant yield and surface tension

The crude biosurfactant dried yield (after acid precipitation) was 1.01 g L^−1^ in the BB medium and 0.77 g L^−1^ in the BU medium, corresponding to a yield per gram of cell dry weight of 0.75 g g^−1^ and 0.81 g g^−1^, respectively (Table 3). Although the BB medium produced a higher yield than the BU medium, the nitrogen sources (beef extract and peptone) in the medium meant it was more expensive than the BU medium, which only contained urea as a nitrogen source. In other studies, crude biosurfactant yields of 0.30–2.3 g L^−1^ have been achieved using a mineral medium supplemented with date molasses and NH_4_NO_3_ as carbon and nitrogen sources [14, 17]. Sousa et al. [23] found 0.44 g L^−1^ of biosurfactant was produced by *B. subtilis* LAMI005 using a mineral medium containing raw glycerol and (NH_4_)_2_SO_4_. In the present study, the amount of biosurfactant (~0.77 g L^−1^) was similar to the values reported by other authors using different substrates.

**Table 3:** Dry cell weight (g L^−1^), crude biosurfactant yield (g L^−1^), oil spreading (cm), emulsification index (%), and surface tension (mN m^−1^) obtained from *Bacillus atrophaeus* 5-2a in BB and BU media

Medium	Dry cell weight (gL^−1^)	Crude biosurfactant yield (gL^−1^)	Oil spreading (cm)	Emulsification index (%)	Surface tension (mN m^−1^)	Yield (g g^−1^)
BB	1.34 ± 0.014a	1.01 ± 0.016a	19.9 ± 0.071a	54.73 ± 0.085b	25.47 ± 0.042b	0.75
BU	0.95 ± 0.028b	0.77 ± 0.014b	19.1 ± 0.10b	59.49 ± 0.33a	26.52 ± 0.057a	0.81

Values are presented as the mean ± standard deviation (*n* = 3). Different superscript letters within a column indicate significant differences (*P* < 0.05) by Duncan’s multiple range test. BB for the fermentation medium used brown sugar, beef extract and peptone as the carbon and nitrogen sources; BU for the optimal medium used brown sugar and inorganic nitrogen urea as the carbon and nitrogen sources. The same as below, unless otherwise specified

The biosurfactants produced using the BB and BU media were able to create low STs of the supernatant, at 25.47 and 26.52 mN m^−1^, respectively (Table 3). The results show that urea is an efficient nitrogen source. There is evidence that the nitrogen source plays an essential part in the biosurfactant production process [26]. Elazzazy et al. [27] showed that urea and NaNO_3_ were the most efficient nitrogen sources for *Virgibacillus salarius* KSA-T; their culture produced a biosurfactant with minimal ST (29.5 mN m^−1^) and maximum emulsifying activity (82 %). Additionally, Ghribi and Ellouze-Chaabouni [28] found that biosurfactant production in their culture was highest using urea. Although there was no significant difference between sodium nitrate, ammonium nitrate, yeast extract, peptone or urea on biosurfactant production, urea was chosen as the cheaper nitrogen source, in comparison to sodium nitrate [21, 25].

#### Emulsifying activity

The emulsifying activity of the biosurfactants produced using the BB and BU media was appreciable, against paraffin oil (Table 3). A significantly higher emulsification index (E_24_, 59.49 %) was obtained using the BU medium compared to the BB medium (E_24_, 54.73 %) (*P* < 0.05). The emulsification properties of a biosurfactant are of practical importance; good emulsification properties increase the potential environmental and industrial applications of biosurfactants [5]. Formation of an oil-in-water emulsion often leads to an improvement in the effective mobility ratio [12]. The cell-free broth produced by the BU medium could probably enhance oil recovery, based on the results observed with paraffin oil.

### Chemical characteristics of the biosurfactants

TLC showed four compounds with Rf values of 0.47, 0.57, 0.75 and 0.8, respectively, when ninhydrin reagent was sprayed, indicating the presence of amino acids. No compounds were observed when sprayed with phenol–sulfuric acid, confirming the absence of sugar moiety. The above results confirm the lipopeptide nature of the biosurfactants. Similar results for other lipopeptide biosurfactants, produced by *B. subtilis*, have been reported [5, 24].

**Fig. 1:**
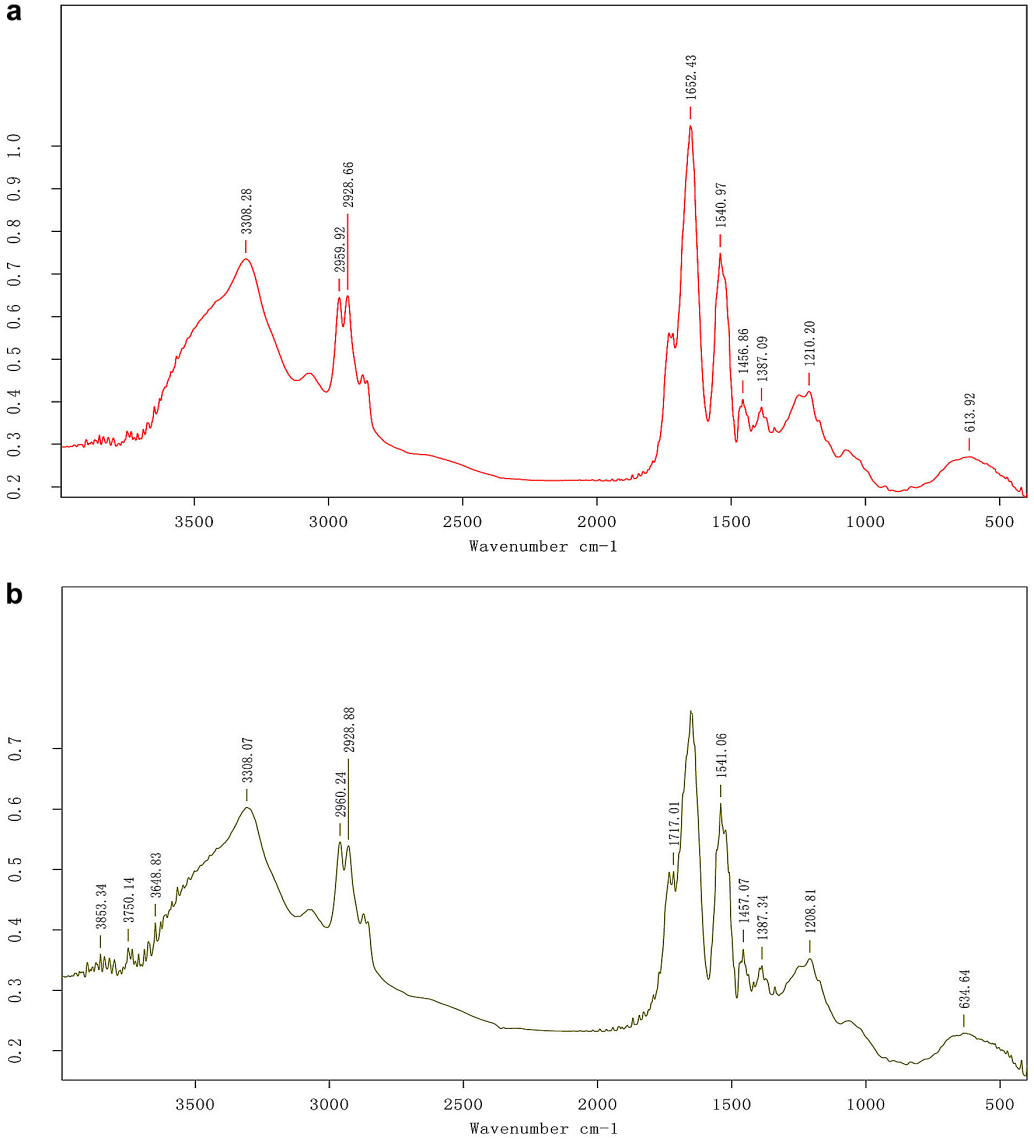
FT-IR absorption spectra of biosurfactants produced by *Bacillus atrophaeus* 5-2a from ‘BB’ (**a**) and ‘BU’ media (**b**). BB for the fermentation medium used brown sugar, beef extract and peptone as the carbon and nitrogen sources; BU for the optimal medium used brown sugar and inorganic nitrogen urea as the carbon and nitrogen sources. The same as below, unless otherwise specified

The FT-IR spectra of the biosurfactants produced by *B. atrophaeus* 5-2a show a characteristic band at 3308.28 cm^−1^, indicating the presence of an –NH bond (Fig. 1). The bands at 1652.40 cm^−1^ and 1540.97 cm^−1^ indicate the presence of the –CO–N bond, while the bands at 2959.92–2928.66 cm^−1^ and 1456.85–1387.09 cm^−1^ reflect the stretch (–CH) of CH_2_ and CH_3_ groups, respectively, in the aliphatic chains. The absorption peak, located at 1736.07 cm^−1^ indicates the presence of ester carbonyl groups (–CO bond). The stretching modes of the –NH, –CO–N and –CO bonds, and the –CH_3_ and –CH_2_ fractions, fall within the same range of wave numbers as previously found; this indicates the similarity in structure of the biosurfactants produced by *B. atrophaeus* 5-2a with lipopeptides previously described in the literature [5, 24].

### Biosurfactant stability

**Fig. 2:**
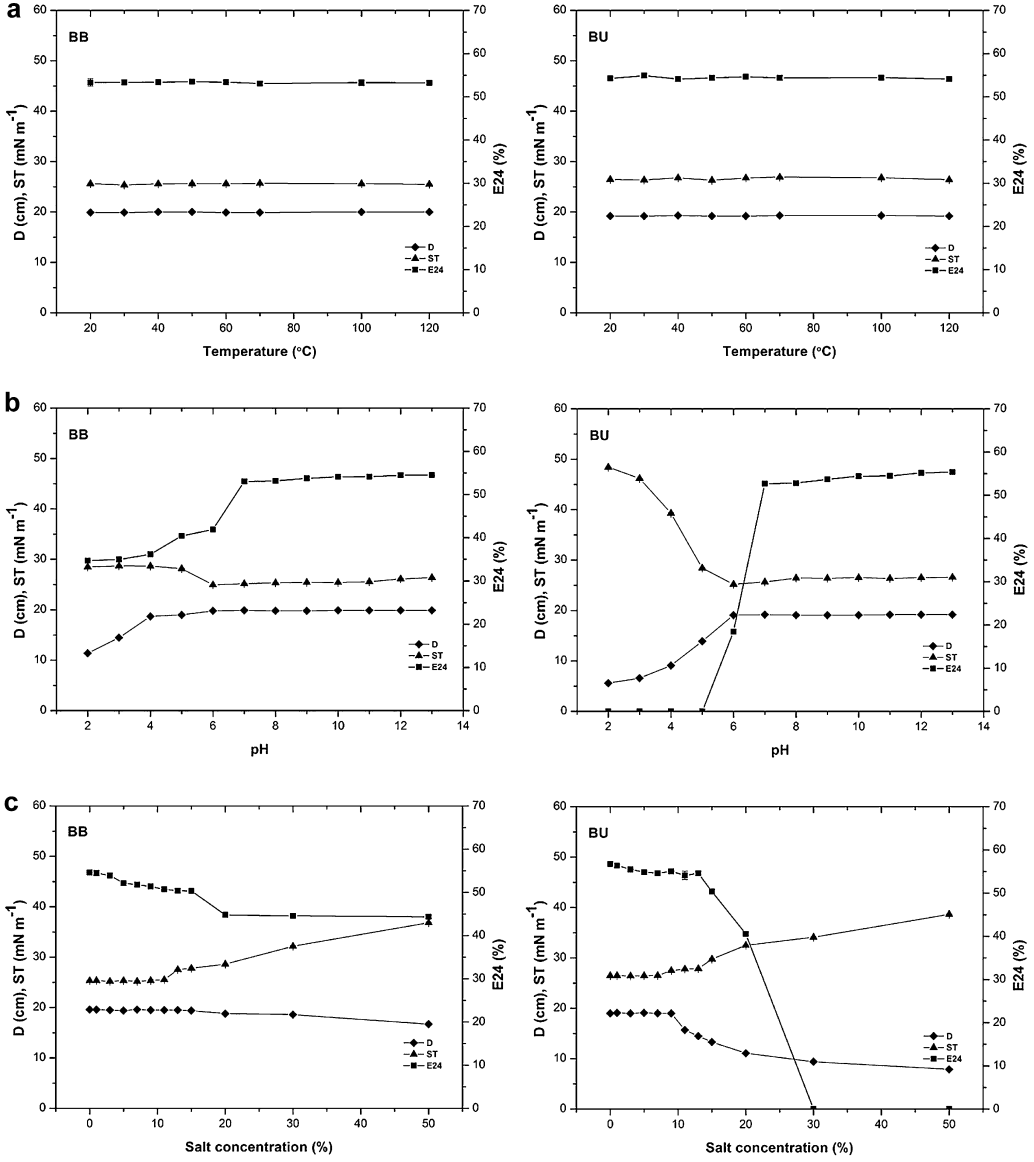
Stability studies for biosurfactants produced by *Bacillus atrophaeus* 5-2a in ‘BB’ and ‘BU’ media, under different conditions of temperature (**a**), pH (**b**), and salinity (**c**). Values represented the mean ± standard deviation (*n* = 3). *D* for oil spreading (cm); *E24* for emulsification index (%); and *ST* for surface tension (mN m^−1^)

Biosurfactants are “green chemicals” used to enhance oil recovery. To use biosurfactants for ex situ MEOR, they need to be stable across a range of temperatures, pH and salinities, to ensure wide applicability [14, 29]. The surface activities of the biosurfactants produced using both the BB and BU media were quite stable over a wide range of temperatures, from 20 to 120 °C (Fig. 2a). Heating the cell-free supernatant up to 100 °C (or autoclaving it at 121 °C) had no significant effect on the surface activity of the biosurfactants. There were no significant differences in the diameter of oil spreading, ST or emulsification activities before and after heating (*P* < 0.05). Several authors have described similar results, in terms of surface activity [30, 31], and performance [14, 29], following heat treatment.

There were minimum deviations in the diameter of oil spreading and ST over the pH range of 6–13, and the emulsification activities of the biosurfactants were stable above pH 7.0. Higher stability was observed under alkaline compared to acidic conditions and the minimum ST was obtained at pH 6.0 (Fig. 2b). Under an acidic pH (pH 2.0 and 5.0) the biosurfactants showed much less activity; the diameter of oil spreading and emulsification index decreased, and the ST increased, due to precipitation of the biosurfactants. These results indicate that increased pH has a positive effect on surface activity and stability of the biosurfactants. Some reports have confirmed the stability of biosurfactants produced by *Bacillus* strains at different pH values, but mostly under alkaline conditions [5, 14].

The surface activity of the biosurfactants produced using both the BB and BU media varied with salinity of 0–50 % (w/v); when the salinity was lower than 9 %, the diameter of oil spreading, emulsification index and ST of the cell-free supernatants were constant. The diameter of oil spreading and emulsification index decreased, and the ST increased, with higher salt concentrations; however, the activity remained high at a salinity of 15 % (w/v). Even at the highest salt concentration (50 %, w/v), the biosurfactants produced in the BB and BU media still had reasonable oil spreading activity and the STs were 36.84 mN m^−1^ and 38.65 mN m^−1^, respectively (Fig. 2c). Overall, relatively high stability, with respect to salinity, was observed in comparison with other studies that used *B. subtilis*, *Nocardiopsis* sp. B4 and *Serratia marscecens* [14, 31, 32].

The biosurfactants produced by *B. atrophaeus* 5-2a were stable over a range of environmental factors and maintained their surface activities. Oil reservoirs are harsh environments, with the potential of high salinity and a wide range of pH values; the observed stability of the biosurfactants assessed in this study, over the pH range of 6–13 and salinity concentrations of 0–15 %, indicates that they would be suitable for oil recovery in most reservoirs. These results show that the biosurfactants from *B. atrophaeus* 5-2a are good candidates for application in MEOR.

### Removal of crude oil from filter papers and sand

**Fig. 3:**
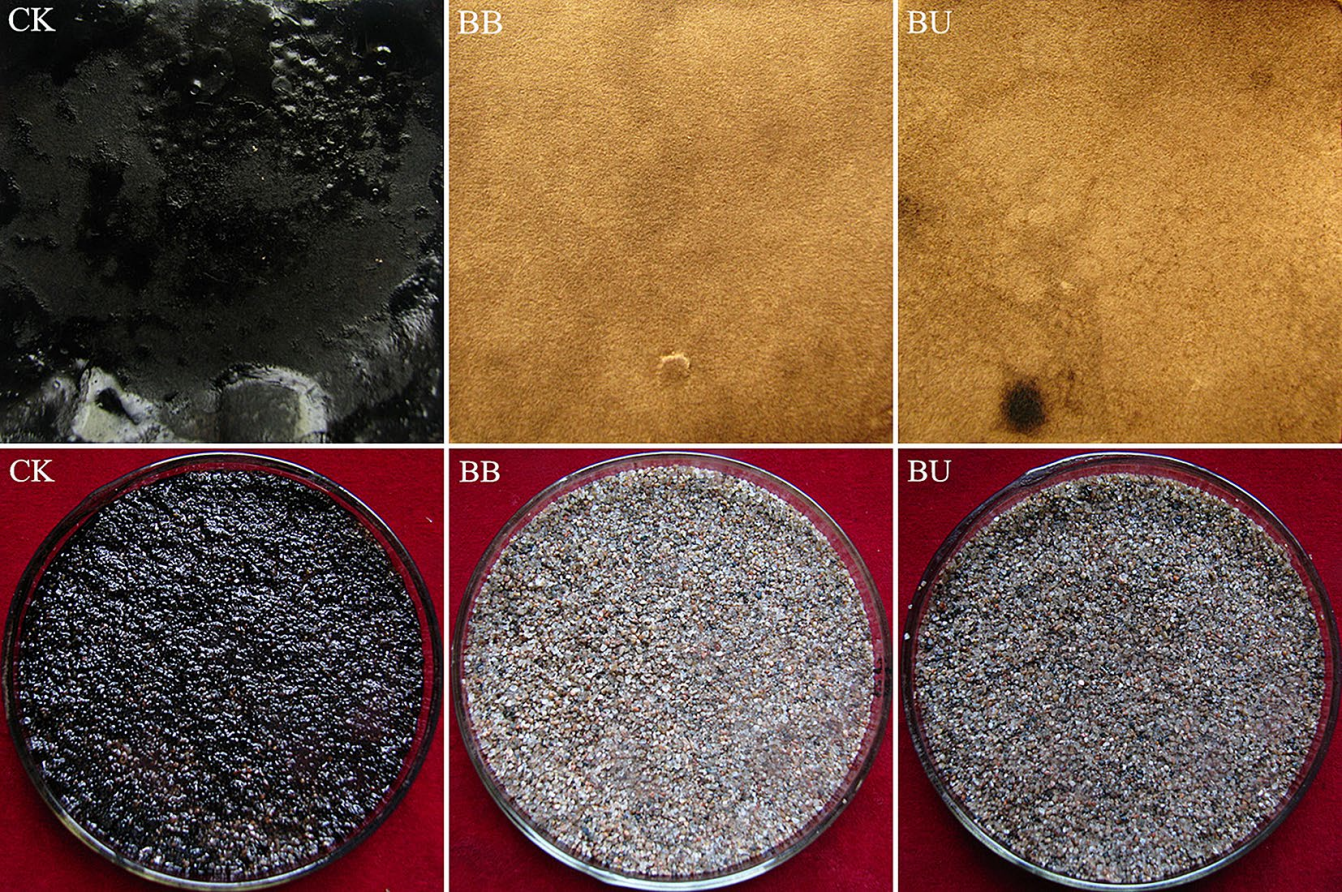
Photos showing the removal efficiency of crude oil adsorbed on filter paper and sand by fermentation both from *BB* and *BU* media

Application of biosurfactants for MEOR is one of the most promising methods for recovering a substantial proportion of residual oil and has been receiving more and more attention recently [12]. Both of the supernatants from the BB and BU media were able to remove the majority of crude oil adsorbed on filter paper and sand (Fig. 3). The removal efficiencies from the filter paper and sand by the supernatant of the BB medium were 94.3 and 94.0 %, respectively; that is, 7.4- and 10.1-fold that of the control. For the supernatant from the BU medium, they were 93.1 and 90.0 %, respectively (7.3- and 9.7-fold that of the control) (Table 4). Pornsunthorntawee et al. [9] reported that 61.6 % of crude residual oil adsorbed in sand was removed using a cell-free broth containing a biosurfactant produced by *B. subtilis* PT2. Pereira et al. [16], who removed crude oil from contaminated sands, found that three strains of *B. subtilis* were effective in oil recovery from sand pores, with rates between 19 and 22 %. The fermentation broths from the present study that contained biosurfactants from *B. atrophaeus* 5-2a were clearly highly efficient in the crude oil removal tests, which is promising for MEOR.

**Table 4:** Crude oil removal efficiencies of fermentation both containing biosurfactants from BB and BU media

Medium	Crude oil removal			
	Filter paper		Sand	
	Removal efficiency (*RE*_*p*_ %)	*RE*_*p*_/*RE*_*Ctrl*_	Removal efficiency (*RE*_*s*_ %)	*RE*_*s*_/*RE*_*Ctrl*_
Ctrl	12.8 ± 0.19c	–	9.3 ± 0.042c	–
BB	94.3 ± 0.049a	7.4	94.0 ± 0.092a	10.1
BU	93.1 ± 0.12b	7.3	90.0 ± 0.057b	9.7

Values are presented as the mean ± standard deviation (*n* = 3). Different superscript letters within a column indicate significant differences (*P* < 0.05) by Duncan’s multiple range test

## Conclusions

*Bacillus atrophaeus* 5-2a produced a potent biosurfactant with high surface activity and emulsification property, when using a cheap mineral salt medium containing brown sugar and urea as the carbon and nitrogen sources, respectively. The biosurfactant was able to reduce the surface tension of the culture supernatant to 26.52 mN m^−1^, and exhibited appreciable emulsification activity against paraffin oil (E24, 59.49 %). The biosurfactants produced by the strain 5-2a from both the BB and BU media remained stable under harsh conditions, including wide ranges of pH, temperature, and salinity. They removed ≥90 % of crude oil from artificially contaminated filter paper and sand. TLC and Fourier transform infrared spectroscopy showed that the biosurfactants produced were a mixture of lipopeptides. This study demonstrated the potential and feasibility of the lipopeptides produced by *B. atrophaeus* 5-2a for application in MEOR. Investigations by laboratory-scale sand-pack columns are warranted to further assess the applicability of the lipopeptides in field applications.

## Methods

### Bacteria, media and oil

Several bacteria were isolated from oil-contaminated surface soils near kowtow machines and oil tanks, adjacent to wells Hua-119 and Hao-129 in Ansai oilfield, Shaanxi province, Northwest China [33]. The oil spreading method was used to select the potential biosurfactant-producing strains, as described by Youssef et al. [34], with minor modifications. Based on its oil spreading activity, *Bacillus atrophaeus* 5-2a was selected for further study; it was identified as *Bacillus atrophaeus* KP314029 by 16S rRNA gene sequencing [33] and was used for the present work. The purified culture was maintained on beef extract peptone agar medium and deposited in the China Center for Type Culture Collection (CCTCC; strain number CCTCC M 2014673).

The basal mineral salt solution (MSS; pH 7.0) used contained (g L^−1^): MgSO_4_·7H_2_O, 0.3; KH_2_PO_4_, 5.0; K_2_HPO_4_·3H_2_O, 5.0; and NaCl, 5.0. The fermentation medium (BB; pH 7.0) used contained (g L^−1^): beef extract, 3.0; peptone, 10.0; NaCl, 5.0; and brown sugar, 10.0.

Crude oil was obtained from a depleted oil well (Hua-20-4) in Ansai oilfield. The oil sample was taken at 1208 m depth in a low-permeability reservoir called Chang 6 (37°04′38 N, 109°02′58 E). The temperature in the reservoir was approximately 40 °C and the well depth reached 1283–1286 m. The oil sample was stored in a plastic bucket at 4 °C until use.

### Effects of carbon and nitrogen sources on biosurfactant production

Biosurfactant production by the culture of *Bacillus atrophaeus* 5-2a was evaluated using a MSS with different carbon and nitrogen sources. Eight carbon source treatments (brown sugar, sucrose, glucose, maltose, starch, mannitol, glycerol and paraffin) were analyzed at final concentrations of 10.0 g L^−1^ in the MSS media, which contained NaNO_3_ (2.0 g L^−1^) and (NH_4_)_2_SO_4_ (1.0 g L^−1^) as the nitrogen sources. Eight nitrogen source treatments were assessed: beef extract, peptone, corn steep liquor, urea, NaNO_3_, NH_4_Cl, (NH_4_)_2_SO_4_ and KNO_3_; each was added to create a final concentration of 3.0 g L^−1^ in the MSS media and brown sugar (10.0 g L^−1^) was used as the carbon source. The initial pH of the media during each treatment was adjusted to 7.0.

To obtain a seed inoculum, the pure culture of *B. atrophaeus* 5-2a was transferred to 100 mL of BB medium and incubated at 30 °C with shaking (120 rpm) for 3 d, creating a cell density of 10^10^ colony-forming units m L^−1^. For each treatment, 5 % seed inoculum was transferred to 600 mL tissue culture vessels containing 100 mL of the treatment medium. The cultures were incubated at 30 °C, with shaking (120 rpm), for 5 days. After fermentation, the samples were collected and the dry cell weight, crude biosurfactant yield, oil spreading, emulsification index and surface tension (ST) were analyzed.

### Effects of the optimal media on biosurfactant production

The ability of the *Bacillus atrophaeus* 5-2a culture to produce biosurfactants was further evaluated using two media. The first was the BB medium, in which the culture presented the best results regarding biosurfactant production. The second medium (hereafter known as BU) used brown sugar and inorganic nitrogen urea as the carbon and nitrogen sources, and was pH 7.0 (g L^−1^): MgSO_4_·7H_2_O, 0.3; KH_2_PO_4_, 5.0; K_2_HPO_4_·3H_2_O, 10.0; NaCl, 5.0; urea, 3.0; and brown sugar, 10.0. The brown sugar and urea as the carbon and nitrogen sources were used to assess the biosurfactant production with an economically viable medium, to test its potential application in MEOR. The cultures were incubated at 30 °C, with shaking (120 rpm), for 5 days. Then, the dry cell weight, crude biosurfactant yield, oil spreading, emulsion index (E_24_) and ST were analyzed.

### Analytical methods

Bacterial cells were harvested by centrifuging (10,000×*g*) for 10 min at 4 °C (Eppendorf, 5804R, Germany) and the dry cell weight (g L^−1^) was determined after drying at 110 °C for 24 h. The cell-free supernatant was taken for the crude biosurfactant yield, oil spreading, emulsion index and ST analyses. Data are expressed as the mean ± standard deviation (*n* = 3). Comparison of group means was conducted using Duncan’s multiple range test (considered significant at *P* < 0.05). The analyses were performed using SAS 9.2 (SAS Institute Inc, Cary, NC, USA). The experiments were performed in triplicate.

#### Oil spreading analysis

Oil spreading analysis tested the displacement activity of the fermentation broth, measured using the method of Youssef et al. [34], with minor modifications. A large plastic tub (25 cm diameter) was filled up with 3000 mL of clean water and two drops of paraffin oil were added to the surface of the water. Then, one drop of fermentation broth was added to the surface of the liquid paraffin. The diameter of the clear zone created on the paraffin oil surface was measured. The larger the diameter of the clear zone, the higher the surface activity of the test solution.

#### Emulsification index

Emulsifying activity was determined by adding 5 mL of paraffin oil to 5 mL of the cell-free supernatant in a glass tube, then mixing it with a vortex for 2 min and incubating it at ambient temperature for 24 h. The emulsification index (E_24_;  %) was calculated as the height of the emulsion layer (mm) divided by the total height of the liquid column (mm) and multiplied by 100 [35]:

$$ E_{24}\,\% = \frac{HE}{HT} \times 100 $$

where HE and HT are the height of the emulsion layer and the total height of liquid column, respectively.

#### Surface tension

ST of the culture supernatants was measured with a digital surface tensiometer (JYW-200A, Chengde, Shandong, China), using the ring method previously described [36]. For calibration, the ST of distilled water was first measured. All ST readings were taken in triplicate and an average value was used to express the ST of each sample.

#### Dried weight measurement of biosurfactants

The biosurfactants were extracted using the acid precipitation method described by Nitschke and Pastore [37]. Briefly, the cell-free supernatant was adjusted to pH 2.0 using 6 M HCl and left overnight at 4 °C for complete precipitation of the biosurfactants. The precipitate was collected by centrifugation (10,000×*g*) for 10 min at 4 °C and washed twice with acidified water (pH 2.0). The crude biosurfactants were oven-dried at 110 °C for 24 h and weighed.

### Characterization of the biosurfactants

#### Thin layer chromatography

The biosurfactants were preliminarily characterized by thin layer chromatography (TLC). The biosurfactant extract (5 mg) was hydrolyzed with 6 M HCl in sealed tubes, maintained at 110 °C for 24 h. The hydrolysate was separated on home-made silica gel plates using CH_3_CH_2_CH_2_CH_2_OH:CH_3_COOH:H_2_O (4:1:1, v/v/v) as the developing solvent system. The compounds separated by TLC were visualized by spraying with ninhydrin 0.5 % (w/v, in water) to identify those with free amino groups. Phenol–sulfuric acid (prepared by mixing 95 mL ethanol, 5 mL of sulfuric acid and 3 g of phenol) was used to identify the sugar moieties. The plates were heated at 110 °C for 5 min until the appearance of the respective colors [5].

#### Fourier transform infrared spectroscopy

The structural groups of the biosurfactants were identified using fourier transform infrared (FT-IR) spectroscopy analysis. The FT-IR spectrum of the dried biosurfactants was recorded on a TENSOR 27 FT-IR spectrometer, equipped with a DLATGS detector (Bruker, Germany); for this, 1 mg of dried biosurfactants was mixed with 100 mg of KBr and pressed down with 7500 kg for 30 s to obtain translucent pellets. The FT-IR spectra, with a resolution of 4 cm^−1^, were acquired between 400 and 4000 wave numbers (cm^−1^).

### Biosurfactant stability

The stability (activity) of the biosurfactants was studied under a wide range of temperatures, pH and salt concentrations [29]. The stability studies were performed using the cell-free supernatant (obtained by centrifugation at 10,000×*g* for 10 min at 4 °C). In the first set of tests, the supernatant was maintained at different constant temperatures, in the range of 20–100 °C for 3 h, and then allowed to cool to ambient temperature. In addition, the supernatant was subjected to autoclave conditions (121 °C, 15 psi for 30 min) as another temperature treatment. In the second set of tests, the pH of the supernatant was adjusted to various pH values, ranging from pH 2 to 13, using HCl (1 N) and NaOH (1 N). In the final set of tests, NaCl was added to the supernatant at different concentrations 0–50 % (w/v). In each series of tests, the diameter of the clear zone, the emulsification index and ST were measured.

### Removal of crude oil from filter paper and sand

The potential use of the biosurfactants for MEOR was assessed using artificially contaminated filter paper and sand. Assessment of the oil removed from artificially contaminated filter paper was carried out using the method of Zhang et al. [38]. For removing the oil from artificially contaminated sand, sand (0.25–0.50 mm fractions) was taken from the Weihe River and 90 g of the sand, contaminated with 10 % crude oil, was transferred to a 600 mL tissue culture vessel containing 150 mL of cell-free supernatant. After 4 days of static incubation at 40 °C in the dark, the mixtures were filtered through sterile cotton wool using washing solution, to separate the sand and crude oil. The crude oil covered sterile cotton wool was extracted with 60 mL hexane, dried by vacuum-rotary evaporation at 40 °C, cooled in a vacuum desiccator to ambient temperature and then weighed (m). Control columns were prepared in the same way, with the addition of 150 mL distilled water. The crude oil removal efficiency (RE_s_ %) was calculated as follows:

$$ RE_{s}\, \%  = \frac{m}{{10}} \times 100 $$

where *m* is the mass (g) of crude oil removed from the artificially contaminated sand after the fermentation broth treatment, and 10 is the original mass of the crude oil.
